# Following in the Footsteps of the Chikungunya Virus in Brazil: The First Autochthonous Cases in Amapá in 2014 and Its Emergence in Rio de Janeiro during 2016

**DOI:** 10.3390/v10110623

**Published:** 2018-11-12

**Authors:** Thiara Manuele Alves de Souza, Edcelha D’Athaide Ribeiro, Valmir Corrêa e Corrêa, Paulo Vieira Damasco, Carla Cunha Santos, Fernanda de Bruycker-Nogueira, Thaís Chouin-Carneiro, Nieli Rodrigues da Costa Faria, Priscila Conrado Guerra Nunes, Manoela Heringer, Monique da Rocha Queiroz Lima, Jéssica Badolato-Corrêa, Márcio da Costa Cipitelli, Elzinandes Leal de Azeredo, Rita Maria Ribeiro Nogueira, Flavia Barreto dos Santos

**Affiliations:** 1 Viral Immunology Laboratory, Oswaldo Cruz Institute, 21040-360 Rio de Janeiro, Brazil; thiara.biomed@gmail.com (T.M.A.d.S.); nandanog@ioc.fiocruz.br (F.d.B.-N.); tatachouin@gmail.com (T.C.-C.); nielircf@ioc.fiocruz.br (N.R.d.C.F.); pricgn@ioc.fiocruz.br (P.C.G.N.); manoelaheringer@yahoo.com.br (M.H.); moniqueq@ioc.fiocruz.br (M.d.R.Q.L.); jessica.badolato@ioc.fiocruz.br (J.B.-C.); marcio_cipitelli@ioc.fiocruz.br (M.d.C.C.); elzinandes@ioc.fiocruz.br (E.L.d.A.); 2 Laboratório Central /Amapa (LACEN/AP), 68908-530 Macapa, Brazil; edcelhamanu@hotmail.com; 3 Laboratório de Fronteira (LAFRON), Oiapoque, 69980-000 Amapa, Brazil; biomedicocorrea@gmail.com; 4 Rio-Laranjeiras Hospital, 22240-000 Rio de Janeiro, Brazil; paulovieiradamasco@gmail.com (P.V.D.); ccih@fblo.com.br (C.C.S.); 5 Gaffrée Guinle University Hospital, Federal University of the State of Rio de Janeiro, 20270-003 Rio de Janeiro, Brazil; 6 Pedro Ernesto University Hospital, University of the State of Rio de Janeiro, 20551-030 Rio de Janeiro, Brazil; 7 Flavivirus Laboratory, Oswaldo Cruz Institute, 21040-360 Rio de Janeiro, Brazil; rita@ioc.fiocruz.br

**Keywords:** chikungunya virus, surveillance, co-infection, ECSA genotype, Asian genotype, Brazil

## Abstract

Currently, Brazil lives a triple arboviruses epidemic (DENV, ZIKV and CHIKV) making the differential diagnosis difficult for health professionals. Here, we aimed to investigate chikungunya cases and the possible occurrence of co-infections during the epidemic in Amapá (AP) that started in 2014 when the first autochthonous cases were reported and in Rio de Janeiro (RJ) in 2016. We further performed molecular characterization and genotyping of representative strains. In AP, 51.4% of the suspected cases were confirmed for CHIKV, 71.0% (76/107). Of those, 24 co-infections by CHIKV/DENV, two by CHIKV/DENV-1, and two by CHIKV/DENV-4 were observed. In RJ, 76.9% of the suspected cases were confirmed for CHIKV and co-infections by CHIKV/DENV (*n* = 8) and by CHIKV/ZIKV (*n* = 17) were observed. Overall, fever, arthralgia, myalgia, prostration, edema, exanthema, conjunctival hyperemia, lower back pain, dizziness, nausea, retroorbital pain, and anorexia were the predominating chikungunya clinical symptoms described. All strains analyzed from AP belonged to the Asian genotype and no amino acid changes were observed. In RJ, the East-Central-South-African genotype (ECSA) circulation was demonstrated and no E1-A226V mutation was observed. Despite this, an E1-V156A substitution was characterized in two samples and for the first time, the E1-K211T mutation was reported in all samples analyzed.

## 1. Introduction

Chikungunya virus (CHIKV) belongs to the *Togaviridae* family, *Alphavirus genus* [1,2,3], and it has been responsible for emerging and reemerging outbreaks in several tropical and temperate regions of the world [4,5]. It is an arbovirus maintained in nature by a transmission cycle involving vertebrate hosts and hematophagous mosquitoes of *Aedes (Ae.)* genus, especially *Ae. aegypti* and *Ae. albopictus* in urban cycles [6,7,8], and humans are the only host capable of developing the clinical forms of the disease.

Chikungunya virus-infected patients may experience an acute febrile illness characterized by severe and debilitating arthralgia [9,10,11] with a sudden onset of high fever (about 40 °C), intense muscle pain in the arms, calves and thighs, as well as, arthralgia in the ankles, elbows, knees, and wrists within the first two weeks of infection [12]. Headache, maculopapular rash, nausea, vomiting, lymphopenia, and moderate thrombocytopenia are also observed [13]. Usually, after two weeks those symptoms are resolved, but a significant portion of patients may experience a chronic disease for months or even years after the initial infection [14,15].

The viral particle is spherical and enveloped, consisting of an icosahedral capsid surrounded by a lipid envelope of approximately 60–70 nm in diameter. The genome consists of a single stranded positive sense RNA of approximately 11.8 kb in length, encoding four non-structural proteins: NSP1–4 and 5 structural proteins: C-E3-E2-6K-E1 [2,3,16]. Four distinct genotypes are described and identified as West African, East-Central-South-African (ECSA), Asian and Indian Ocean Lineage (IOL). The IOL emerged as an ECSA monophyletic group [17,18] due to an E1-A226V mutation associated with an increase in *Ae. albopictus* transmission of the virus, which caused several outbreaks in the Indian Ocean, parts of India, Singapore, Malaysia, Thailand, Sri Lanka, Gabon, and Italy since 2006 [19].

In Brazil, chikungunya autochthonous cases were confirmed when two CHIKV genotypes were independently identified during 2014 in Oiapoque (Amapá, AP) and originating from the Caribbean and South America strains, and the ECSA genotype in Feira de Santana (BA), originating from Angola (West Africa) and Asia [17]. The virus rapidly spread, causing large epidemics in urban areas [20] and co-circulating with the newly introduced Zika virus (ZIKV) and the four endemic dengue virus (DENV) serotypes. In areas where those arboviruses co-circulate, suspected cases may be misdiagnosed.

Dengue and zika may be presented with mild arthralgia and rash, while chikungunya and dengue may be initially clinically undistinguished due to the high fever, for instance. Therefore, clinical differential diagnosis may be troublesome. This scenario can be worsened by the unavailability of reliable tests for the laboratorial diagnosis [5,21].

The state of Rio de Janeiro (RJ), in the southeast region of Brazil, has been a hotspot for DENV introduction and spread since the 1980s. Oiapoque, in the northern state of Amapá (AP), was the epicenter for the CHIKV epidemic during its first introduction in 2014. From 2015 to 2017, almost 500,000 chikungunya cases were reported in Brazil, 277,882 in 2016 alone [22]. Here, we aimed to investigate chikungunya suspected cases and the possible occurrence of coinfections among three circulating arboviruses from epidemics that occurred in AP (north region) during 2014 and RJ (southeast region) during 2016. We further performed the genotyping and molecular characterization from the partial sequencing of CHIKV representative strains. Considering that mutations such as A226V in the ECSA genotype have not yet been observed in the Brazilian strains, molecular characterization studies can represent an important tool to monitor the dispersion of circulating genotypes and identify possible mutations that facilitate mosquitoes’ transmission.

## 2. Material and Methods

### 2.1. Ethical Statement

The cases analyzed in this study were from projects approved by the Oswaldo Cruz Foundation Ethic Committee (CAAE 57221416.0.1001.5248 and CAAE 30757314.1.0000.5248). All patients enrolled signed an informed written consent. The patients’ personal information was anonymized before the data was accessed. This study accessed the patients’ information on demographic characteristics, physical signs, and symptoms.

### 2.2. Clinical Samples

The biological samples analyzed in this study were collected during arboviruses epidemics (CHIKV and/or DENV and/or ZIKV) that occurred in representative cities of the north and southeast regions of Brazil. In the North region, serum, plasma and/or whole blood samples (*n =* 208) were collected from CHIKV suspected cases during an active surveillance performed by the Laboratório Central do Estado do Amapá (LACEN/AP), Laboratório de Fronteira do Oiapoque (LAFRON/AP), and by the Flavivirus Laboratory Regional Reference Center for Dengue, Zika, Chikungunya, and Yellow Fever (LABFLA, Oswaldo Cruz Institute, FIOCRUZ) in representative cities of the Amapa state (Oiapoque, Aporema, Porto Grande, Macapá, Santana and Mazagão) from 2014 to 2015. In the southeast region, samples of serum, plasma, and whole blood from suspected cases of CHIKV and/or DENV and/or ZIKV were collected during a cross-sectional and observational study performed by the Laboratory of Viral Immunology, (LIV, IOC/FIOCRUZ) in RJ (*n =* 91), from April 2016 to May 2016. Patients were attended at Rio Laranjeiras Hospital (HRL, Rio de Janeiro), where an infectious disease physician collected data on demographic characteristics, symptoms, and physical signs using a structured questionnaire. The inclusion criteria covered patients in any age group and any gender who experienced a febrile illness accompanied by intense polyarthralgia according to the Ministry of Health [5]. Exclusion criteria were those patients who did not agree to participate in the study or who were suspected of other infections.

### 2.3. Arboviruses Laboratorial Diagnosis

All cases were screened for DENV, ZIKV, and CHIKV as a differential diagnosis. Acute samples (≤7 days after onset of symptoms) were analyzed by molecular diagnosis and convalescent ones (>7 days after the onset of symptoms) by serological methods.

Chikungunya infection in acute samples was determined by using the RT-PCR protocol described elsewhere [23]. The Simplexa™ CHIKV kit (Focus Diagnostics, Cypress, CA, USA) for virus detection and the chikungunya non-structural protein 2 (NSP2) standard kit (Genesig^®^, Camberley, UK) for quantification of CHIKV confirmed cases were used as complementary strategies according to the manufacturers’ instructions. The determination of anti-CHIKV IgM antibodies was performed according to the in-house IgM capture ELISA protocol described by the CDC and the Ministry of Health (2014) and by using the anti-CHIKV ELISA IgM (Euroimmun, Lubeck, Germany) according to the manufacturer’s protocol. The IgM antibody detection using ELISA immunofluorescent assays is a promising and highly reliable approach to be used during the active surveillance of CHIKV as it represents recent and active infections, and may be detectable after 2 days of disease [24,25].

For dengue serological diagnosis, suspected cases were submitted to the Dengue Virus IgM Capture DxSelect™ (Focus Diagnostics, Cypress, CA, USA) and Platelia™ Dengue NS1 Ag ELISA (BioRad Laboratories, Hercules, CA, USA). Molecular detection and DENV serotype identification were performed by conventional RT-PCR as described previously by Lanciotti et al. [26] and by the real-time RT-PCR Jonhson et al. [27]. The viral RNA was extracted using the QIAamp Viral RNA Mini kit (Qiagen, Hilden, Germany) following the manufacturer’s instructions and stored at −70 °C. Aiming to further exclude DENV infection in negative cases, all samples were further tested using the Simplexa™ Dengue Real Time RT-PCR (Focus Diagnostics, Cypress, CA, USA) according to the manufacturer’s protocol, for viral qualitative detection and typing of DENV. Due to the cross-reactivity among flaviviruses in the serological methods, suspected zika cases were tested by the real-time RT-PCR for ZIKV, as described previously by Lanciotti et al. [28]. All samples were also subjected to a RT-PCR for detection of alphaviruses (Mayaro virus–MAYV) according to de Morais Bronzoni et al. [29].

### 2.4. Statistical Analysis

Statistical analysis was performed using SPSS Software 21.0 version (IBM, New York, NY, USA). The chi-squared test was used to evaluate differences between groups. Values of *p* < 0.05 were considered significant for all statistical analysis.

### 2.5. Chikungunya Virus Genome Amplification, Sequencing, and Phylogenetic Analysis

Chikungunya positive cases by qRT-PCR were randomly selected for partial sequencing (E1 gene) according to the semi-nested protocol described by Reference [30] and phylogenetic analysis. This protocol amplifies approximately 469 nucleotides along the E1 gene, and due to sequence editing losses, we aimed to amplify approximately 375 nucleotides along this region. This fragment allows us to answer the questions about this study, which consists of the genotyping of circulating strains and observation of specific modifications, such as the A226V mutation, which allowed the emergence of the Indian Ocean Lineage (IOL). Viral RNA was extracted from serum, plasma or whole blood of CHIKV cases using the QIAamp Viral RNA Mini kit (Qiagen, Hilden, Germany), following the manufacturer’s protocol. The fragments generated were purified using PCR Purification Kit or Gel Extraction Kit (Qiagen, Inc., Germany) and sequenced in both directions using the BigDye Terminator Cycle Sequencing Ready Reaction version 3.1 kit (Applied Biosystems^®^, Foster City, CA, USA). The thermocycling conditions consisted of 40 cycles of denaturation (94 °C/10 s), annealing (50 °C/5 s) and extension (60 °C/4 min). Sequencing was performed on an ABI 3730 DNA Analyzer, Applied Biosystems^®^, California, USA. The sequences analysis was performed using BioEdit (http://www.mbio.ncsu.edu/bioedit/bioedit.htmL), sequences’ identity was performed using BLAST (http://blast.ncbi.nlm.nih.gov/Blast.cgi) and alignments using CLUSTAL OMEGA (http://www.ebi.ac.uk/Tools/msa/clustalo/). The data set was constructed with sequences previously deposited on GenBank and representative of each genotype and with sequences identified using BLAST. Phylogenetic trees were constructed using the MEGA 6 (http://www.megasoftware.net/), by the “neighbor-joining” method and maximum-likelihood, Kimura-2 parameter model (K2), with a bootstrap of 1000 replications. Both methods were used as confirmation of the results. The trees were built based on the analysis of the best-fit-for model, as provided by the software. Sequences available in GenBank (https://www.ncbi.nlm.nih.gov/genbank/) were used as reference for the different genotypes of CHIKV (Asian, ECSA and West African), Table 1. Partial CHIKV genome sequences were deposited in GenBank.

## 3. Results

### 3.1. Laboratorial Investigation of CHIKV Suspected Cases in Amapa, 2014–2015

To investigate CHIKV cases in the north region of Brazil, suspected cases (*n =* 208) were collected from the epidemic occurring during 2014 and 2015 in the municipality of Amapa (AP) Brazil. Acute (48.8%, 100/208) and convalescent cases (51.9%, 108/208) were analyzed.

Anti-CHIKV IgM was detected in 48.1% (52/108) of the convalescent cases, independently of the test used. Considering the *in-house* CDC IgM ELISA, 36.5% (19/52) of the cases tested were confirmed, whereas 46.1% (24/52) were confirmed using the commercial kit. Only 17.3% (9/52) of the cases were simultaneously confirmed by the two methodologies. Overall, of the 44 cases, dengue NS1 was detected in 18.0% and anti-DENV IgM in 61.0%, while both dengue NS1 and anti-DENV IgM were detected in 20.0% of cases.

Molecular diagnosis on acute samples confirmed 53.0% (53/100) of the CHIKV suspected cases tested. The qRT-PCR for CHIKV detection confirmed 11.3% (6/53) of the positive cases, whereas the commercial Simplexa™ CHIKV kit confirmed 30.1% (16/53). Positive cases confirmed simultaneously by the two methodologies were 58.5% (31/53). A total of 66.0% (35/53) of the confirmed CHIKV cases were randomly selected and quantified, and viral titers ranged from 1.01 × 10^2^ to 9.84 × 10^3^ copies of RNA/μL. 

Exceptionally, eleven acute cases on the 6th and 7th day of symptoms were also submitted to serological tests for anti-CHIKV IgM detection. The results showed that 36.3% (4/11) presented a positive result to anti-CHIKV IgM. Moreover, two of those cases were negative by qRT-PCR.

As a differential diagnosis, all CHIKV suspected acute cases (*n =* 100) were also submitted to DENV diagnosis by RT-PCR and, 10 were positive (8 DENV-1 and 2 DENV-4). Two of those cases were also positive for DENV NS1 and to anti-DENV IgM.

Co-infections were considered by positive results on RT-PCR methodologies. Chikungunya virus monoinfections were identified in 51 out of 61 (83.6%) acute cases positive by RT-PCR, DENV-1 monoinfections were characterized in 11.4% (7/61), and DENV-4 monoinfections in 1.6% (1/61) of the positive cases. One case was characterized as CHIKV/DENV-1 co-infection and one as CHIKV/DENV-4 one, see Table 2.

Although we performed statistical analysis on the monoinfected and coinfected groups, we did not consider the significance observed in the variables of age, and the symptom of cough, due to the small number of coinfected cases analyzed (only two patients).

#### Clinical and Epidemiological Aspects of Monoinfections and Coinfections in Amapá

In this study, CHIKV monoinfections were reported in Macapá (9.8%), Oiapoque (88.2%), and Porto Grande (1.9%). The DENV-1 monoinfections were reported in Macapá (42.8%) and Oiapoque (57.1%), and one DENV-4 monoinfection in Macapá. The CHIKV/DENV-1 co-infection was reported from Macapá and the CHIKV/DENV-4 from Oiapoque, see Table 2. Females were more affected than males in CHIKV monoinfection cases (72.5% and 27.4%, respectively), while the opposite occurred in DENV-1 monoinfection cases (14.2% and 85.7%, respectively). The DENV-4 monoinfection case occurred in a male patient, while the CHIKV/DENV-1 and CHIKV/DENV-4 coinfections occurred in females.

In CHIKV monoinfections, a higher frequency was observed in individuals aged 46–50 (13.7%), followed by 26–30 (11.8%), 11–15 (9.8%), 16–20 (9.8%), and 36–40 (7.8%). In 19.6%, the patient’s age was not available. The DENV-1 monoinfections, on the other hand, showed higher frequencies in patients aged 31–35 (28.6%) and 36–40 (28.6%). The DENV-4 monoinfection occurred in a child with <10 years old. The CHIKV/DENV-1 coinfection occurred in an individual aged 41 to 45 years. The case of CHIKV/DENV-4 coinfection occurred in an individual aged 31 to 35 years, see Table 2.

Considering the frequency of clinical manifestations in CHIKV confirmed cases (*n* = 56), the results show that arthralgia, chills, headache, rash, fever, lower back pain, myalgia, nausea, pruritus, and prostration are among the most common signs or symptoms in all groups of patients, see Table 2.

### 3.2. Investigation of CHIKV Suspected Cases in Rio de Janeiro during 2016

To investigate CHIKV cases in the southeast region of Brazil, 91 samples of plasma, serum or whole blood were collected in RJ. Of those, 78.2% (71/91) were classified as acute (≤7 days after onset of symptoms) and 21.9% (20/91) as convalescent cases (>7 days after onset of symptoms).

The results demonstrate that qRT-PCR confirmed 64.8% (59/91) cases for CHIKV. Of those, 79.6% (47/59) had up to seven days after onset of symptoms, 15.2% (9/59) between eight and 15 days after onset of symptoms, 1.6% (1/59) 20 days of disease, 25 days of disease and curiously, 71 days of disease. Among the 59 CHIKV cases confirmed by qRT-PCR, 37.3% (22/59) were randomly selected and quantified, and the viral quantification ranged from 1.07 × 10^0^ to 9.51 × 10^0^ copies of RNA/μl.

Of the confirmed CHIKV cases by qRT-PCR, 39.0% (23/59) also presented serological evidence by the results obtained using an anti-CHIKV ELISA IgM test. Of those, 43.5% (10/23) cases had more than seven days after onset of symptoms, including a case with 71 days of disease. In addition, 12.0% (11/91) cases presented CHIKV serological evidence using the anti-CHIKV ELISA IgM kit, being 18.1% (2/11) with four days of disease, 9.0% (1/11) with five days of disease, 9.0% (1/11) with six days of disease, 27.2% (3/11) with seven days of disease, and 36.3% (4/11) with more than seven days of disease.

As a differential diagnosis, all suspected CHIKV cases (*n* = 91) were submitted to serological and/or molecular methodologies for exclusion of dengue and zika. No case was detected by RT-PCR for detection and typing of DENV. The Simplexa™ dengue kit confirmed 4.3% (4/91) cases as belonging to DENV-4 serotype, being 3/4 (75.0%) acute cases and 1/4 (25.0%) with 10 days of disease. Of those, 1/4 (25.0%) was also detected as DENV-4 by the qRT-PCR established by Johnson et al. (2005). In addition, 26/91 (28.6%) cases were confirmed for ZIKV by the qRT-PCR, with 22/26 (84.6%) acute cases with up to six days of disease, 1/26 (3.8%) with eight days, 1/26 (3.8%) with 13 days, 1/26 (3.8%) with 15 days, and 1/26 (3.8%) with 25 days of disease.

By RT-PCR, 69/91 (75.8%) of the cases were confirmed as monoinfections or coinfections by CHIKV and/or DENV and/or ZIKV. From those, 40/69 (58.0%) were characterized as CHIKV monoinfections, 9/69 (13.0%) as ZIKV monoinfections, 16/69 (23.2%) as CHIKV and ZIKV coinfections, 3/69 (4.3%) as CHIKV/DENV-4 coinfection, and one (1/69, 1.4%) as a ZIKV/DENV-4 coinfection.

#### Clinical and Epidemiological Aspects of Monoinfections and Coinfections in Rio de Janeiro

Female patients were more affected than males in CHIKV monoinfection cases (55% and 45%, respectively) and CHIKV and ZIKV coinfections (62.5% and 37.5%, respectively), with a prevalence of 100% in CHIKV and DENV-4 coinfections (100%) and ZIKV and DENV-4 coinfections (100%), Table 3.

In CHIKV monoinfections, individuals 26 to 30 years old (22.5%) were the most affected, followed by 51–55 years old (15.0%), 56–60 years old (12.5%), and >65 years old (12.5%). The ZIKV monoinfections were observed in individuals between 26 to 30 years old (22.2%), 36 to 40 years old (22.2%), and 41 to 45 years old (22.2%). The CHIKV/ZIKV coinfections were confirmed in individuals in the age group of 41–45 years old (25.0%), followed by 61–65 years old (12.5%), and >65 years old (12.5%). The CHIKV/DENV-4 coinfections were reported in individuals aged between 16 and 20 years old (33.3%), 21–25 years old (33.3%), and 56–60 years old (33.3%). The ZIKV/DENV-4 coinfection occurred in a 51-year-old patient. Arthralgia, chills, headache, exanthema, fever, lower back pain, myalgia, nausea, pruritus, and prostration were among the most common signs and symptoms observed in all CHIKV confirmed cases groups (*n* = 56), see Table 3. No significance was observed between monoinfected and coinfected cases in any of the variables tested. Considering the signs and symptoms presented, arthritis was significantly more frequent in the monoinfected cases when compared to the coinfected ones (*p* = 0.042).

### 3.3. Phylogenetic Analysis and Molecular Characterization of CHIKV Representative Strains of Amapá, AP, and Rio de Janeiro, RJ, Brazil

**Figure 1:**
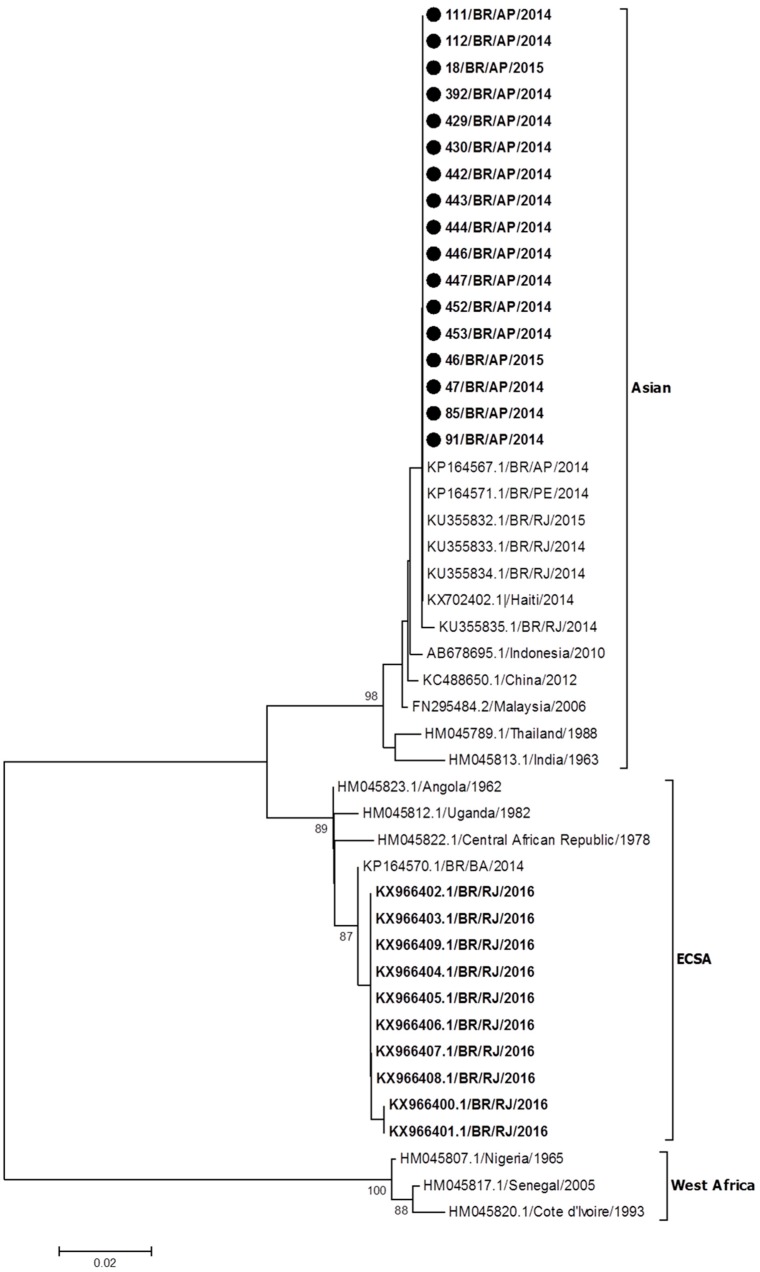
Phylogenetic analysis based on 375 nucleotides recovered from the E1 gene of CHIKV strains identified in Rio de Janeiro (*n* = 10) during 2016 and in Amapa (*n* = 17). Neighbor-joining method, Kimura-2-parameter model (K2), bootstrap of 1000 repetitions. The analyzed CHIKV sequences are represented by a black circle. The CHIKV strains were designated as follows: GenBank accession number (or strain name/country/year). ECSA: East-Central-South African genotype.

Phylogenetic analysis was possible by the recovery of 375 nucleotides from the partial sequencing of the E1 gene from representative CHIKV strains detected during the epidemics that occurred in RJ (*n* = 10) during 2016 [30] and in AP (*n* = 17) during 2014–2015 (this study). Reference sequences available from GenBank were used representing the Asian, ECSA, and West African genotypes. After comparative analysis, the results showed that all strains from AP were grouped in the branch of the Asian genotype, while those from RJ clustered in the branch of the ECSA genotype, together with the sequence of a sample identified in Bahia in 2014 (Figure 1).

Sequences from this study were deposited in GenBank under accession numbers showed in Table 4. 

Molecular characterization was also performed and no amino acid differences were observed in the AP strains. Despite this, partial analysis of the E1 fragment of the RJ strains did not demonstrate the A226V mutation, revealing that the amino acid alanine was present in the E226 position. Interestingly, a K211T amino acid substitution was identified in all samples analyzed and a V156A substitution was identified in two samples from this study. It is important to note that the CHIKV sequences identified in BA belonging to the ECSA genotype did not show this substitution at amino acid 211, thus having a lysine (K), which was also observed in the reference sequence from Angola (1962).

## 4. Discussion

The present study investigated CHIKV suspected cases in Brazil from patients experiencing a febrile illness accompanied by intense polyarthralgia during outbreaks that occurred in the north region (Amapá, AP) in 2014, characterized as the first autochthonous cases, and in the southeast (Rio de Janeiro, RJ) in 2016, after its spread to the southeast region. Due to the epidemiological situation presented and the co-circulation of arboviruses (CHIKV and DENV) in the north region during the period, surveillance studies, such as the one presented here, play a role in understanding CHIKV’s spread in Brazil.

In AP, the majority of the CHIKV cases analyzed here were from the city of Oiapoque (27/107; 25.2%), the epidemic epicenter. Moreover, 865 chikungunya cases were reported in the entire state of AP in 2015; however, from those, 789 cases were reported only in the city of Oiapoque [36].

More than half of the acute and convalescent cases (107/208; 51.4%) analyzed in AP were confirmed by molecular and/or serological methodologies. The combined use of serological and molecular tests in the acute phase diagnosis of CHIKV infection may be recommended, since previous studies have demonstrated that anti-CHIKV IgM antibodies can be detected from two days of disease [24,25].

The commercial kit anti-CHIKV ELISA IgM (Euroimmun) used in this study is recommended by the Brazilian Ministry of Health and has 98.1% sensitivity and 98.9% specificity, according to the manufacturer. The Platelia™ Dengue NS1 Antigen kit is used for the early diagnosis of dengue infections based on NS1 antigen capture, and as a serology test, presents higher sensitivity and specificity considering the IgM and IgG detections. Anti-DENV IgM is detected between three to five days after the onset of infection and IgG, after 10 to 14 days, different from the NS1 antigen which can be detected from 0 to 9 days after the onset of symptoms, with a peak on six to 10 days of infection [37]. According to the manufacturers, the Panbio dengue IgM Capture ELISA and Dengue Virus IgM Capture DxSelect commercial kits used here present sensitivities of 94.7% and 96.0% and specificities of 100% and 97%, respectively. Moreover, the manufacturers’ sensitivities reported for both NS1 capture kits used in this study (Platelia ™ Dengue NS1 Ag-ELISA and Dengue NS1 Antigen DxSelect ™) are of 95.0% and 88.2%, and specificities of 100%.

In AP, 13.0% (13/100) of acute samples were confirmed for DENV by molecular and/or serological techniques, and among those confirmed by RT-PCR (*n* = 10), eight were identified as DENV-1 and 2 as DENV- 4. Of the convalescent samples, 40.7% (44/108) were confirmed for dengue using serological methodologies. Thus, the data demonstrated that during 2014–2015, despite distinct arboviral epidemics occurring simultaneously in AP, chikungunya was predominant and only a few dengue cases were reported. In Laos in 2014, an opposite scenario was characterized during an epidemic, and a higher percentage of dengue cases was reported when compared to chikungunya [38].

In 2016, until epidemiological week 52, RJ reported 85,200 cases of chikungunya [22] and the spread of the virus throughout the Brazilian territory, including RJ, was stressed due to the high levels of mosquito vectors infestation throughout the country [39]. The results from the hospital-based study performed here, most of the cases analyzed were confirmed for CHIKV (70/91; 76.9%) by molecular and/or serological methodologies, with the majority detected by RT-PCR (50/70, 71.4%) in acute samples. Despite this, CHIKV viral RNA was also detected in 18 convalescent samples with more than 10 days of symptoms, and curiously, one of those was collected after 71 days of disease. In humans, the persistence of CHIKV viral RNA in perivascular macrophages of synovial fluid in a chronic patient has been demonstrated for up to 18 months after infection and this may be justified by T cell depletion due to the strong immune response during the acute phase, causing as a consequence the viral persistence [40]. Other studies have demonstrated long viraemia in specimens collected from six days after onset of symptoms [41] and persistence of viral RNA and/or genomic products after 17 days of disease [42]. Further studies are needed to elucidate how and for how long CHIKV persists, as well as its relationship with the patient’s immune system and clinical evolution for chronic arthralgia [43]. To date, it has been demonstrated that viral persistence is directly associated with immune inefficiency and efficient viral escape [40].

All acute phase samples from AP and RJ were quantified by qRT-PCR and the results demonstrated low viral titers, which does not corroborate with the high viremia observed during this infection, which can increase from 10^9^ to 10^12^ copies of RNA/mL [44,45]. The low viral titer obtained here may be due a test limitation or sensitivity. Although higher, there are no major differences between the high viremia in acute symptomatic patients when compared to the asymptomatic patients [42].

The first cases of ZIKV in Brazil in 2015 led to the occurrence of a triple epidemic of arboviruses caused by the co-circulation of CHIKV, DENV, and ZIKV in some states of the country. This situation did represent a serious public health problem due difficulties in diagnosing the cases clinically due to their similar signs and symptoms and due to the lack of reliable tests for the differential laboratorial diagnosis [21,46]. The combination of laboratorial methods, such as the strategy used here, despite not always used in most laboratories due to its cost, is important for the accurate case confirmation. In this study, co-infections by CHIKV/DENV and CHIKV/ZIKV were observed in both states and this observation has been described previously in areas where multiple arboviruses circulate in the Americas, Africa, and Asia [38,47,48,49]. 

A higher frequency of CHIKV infections was observed in females in both Brazilian states studied. Moreover, although the virus affect both sexes, females are more likely to present a more severe disease and persistent joint pain [50,51]. Individuals over 15 years old were more frequently affected. In AP, the highest number of cases was reported in individuals between 16 and 20 years and between 26 and 55 years. In RJ, the highest frequency of confirmed cases was in the age group of 26 to 30 years, followed by 41 to 45 and 51 to 55 years old. These data corroborate previous studies indicating the predominance of adults in the CHIKV-affected population, such as observed during the Réunion Island epidemic in 2006 [52]. Furthermore, it has been shown that older age groups are at risk for a more severe disease and persistent arthralgia due to the presence of comorbidities and a less efficient immune response. In fact, individuals over 65 years old presented a 50-fold higher mortality rate when compared to individuals 45 years old and under [50,51].

Fever, arthralgia, myalgia, prostration, edema, exanthema, conjunctival hyperemia, lower back pain, dizziness, nausea, retroorbital pain, and anorexia were the predominating signs and symptoms reported by the chikungunya cases analyzed here and have been described as very common ones during CHIKV infections [52,53,54,55,56]. Arthralgia is the most characteristic symptom of chikungunya, is polyarticular, and may persistent up to 18 months after the acute phase [52]. Other studies reported persistent arthralgia in several CHIKV epidemics [50,55,57,58,59,60,61]. One limitation of the study is that no information is available in how many cases evolved to a chronic phase in both states analyzed. During the chronic phase of the disease, disabling pain can affect the patient’s daily activities [52], causing a reduction in quality of life, asthenia, depression, and anxiety [61,62]. Thus, it is important to monitor the patient’s medical condition during this phase, to treat emotional symptoms [62,63]. 

Finally, this study also performed the phylogenetic analysis and molecular characterization of representative strains of CHIKV from AP and compared to those from RJ, published previously by our group. In the AP, although no amino acids substitutions were observed, it was demonstrated that all strains analyzed belong to the Asian genotype, corroborating other studies that revealed the city of Oiapoque (AP) as the introduction site for this genotype in Brazil during 2014, originating from the Caribbean and South America [17,64,65]. 

Nunes et al. [17] observed two separate CHIKV introductions in Brazil and reported the emergence of the Asian and ECSA CHIKV genotypes, with possible different dynamics of the genotypes dispersion across the country. The ECSA genotype was introduced from Angola to Feira de Santana, Bahia (BA) in June of 2014, when it was introduced independently from strains originating in Angola (West Africa). The Asian genotype circulating in the Caribbean and South America was introduced in Oiapoque in early September of 2014, probably imported from French Guiana, a border country. Due to the similarities with DENV transmission, it was suggested that CHIKV would have the same dynamics, with transmission peaks occurring between January and April. Furthermore, it would be expected a prevalence of the Asian genotype in the tropical regions of Brazil, where *Aedes aegypti* is well established, and the ECSA genotype in subtropical and temperate regions, where *Aedes albopictus* is more abundant. In the long run, both genotypes could disappear from the region if the population immunity levels increased sufficiently. Alternatively, CHIKV could establish an enzootic cycle in the region with sporadic human epidemics. This pattern has been observed in Africa and potentially in Southeast Asia, where there is some evidence of a transmission cycle of the causal virus involving non-human primates and mosquitoes inhabiting the forest similar to that observed for wild-type yellow fever virus.

In RJ, the ECSA genotype circulation was demonstrated for the first time during an outbreak in 2016. In addition, the molecular characterization revealed that an alanine was present at position E1_226_ gene, showing no A226V mutation in the strains from RJ. Studies carried out during the 2005–2006 epidemic on the Réunion Island revealed that this mutation was responsible for the emergence of the IOL variant, responsible for the increased transmission of CHIKV by *Aedes Albopictus* [66,67].

Despite this, this study revealed a substitution on E1 (K211T) in all samples analyzed and on E1 (V156A) in two samples. The K211T substitution has not yet been identified in the strains from Bahia, which has a Lysine (K), such as the reference sequence from Angola (1962). Other studies are needed to clarify the consequences of those changes in the mosquitoes fitness and in the human immune system, but some publications suggest that new mutations such as L210Q, I211T, and G60D on the E2 gene from the IOL may also offer advantages for CHIKV transmission by *Aedes Albopictus* [66,68,69]. For *Aedes aegypti*, the E1 (K211E) and E2 (V264A) mutations were observed in CHIKV strains from India during 2006–2010 [70,71].

It is important to note that the E1 gene represents a target region for this analysis due to its high antigenic variability and which is also required for binding, virus entry into target cells, and viral replication during infection [1,2]. 

## 5. Conclusions

The exponential growth of chikungunya, especially in populated and touristic regions, such as RJ, represents a serious public health problem and the co-circulation of three arboviruses (DENV, CHIKV, and ZIKV) as observed here, does impair the laboratory and clinical diagnosis, making it a challenge. Moreover, monitoring the dispersion of CHIKV genotypes and identifying possible mutations that facilitate the viral transmission by mosquitoes vectors is of primary importance, especially in large regions with high vector densities and presence of susceptible individuals such as those observed in Brazil. 

## Figures and Tables

**Table 1:** GenBank available sequences used for phylogenetic analysis representative of the different chikungunya virus (CHIKV) genotypes (Asian, East-Central-South African (ECSA), and West African).

Genotype	Location/Year	GenBank Accession Number	Reference
Asian	Amapa/Brazil/2014	KP164567.1	[17]
	Pernambuco/Brazil/2014	KP164571.1	[17]
	Rio de Janeiro/Brazil/2015	KU355832.1	[31]
	Rio de Janeiro/Brazil/2014	KU355833.1	[31]
	Rio de Janeiro/Brazil/2014	KU355834.1	[31]
	Rio de Janeiro/Brazil/2014	KU355835.1	[31]
	Haiti/2014	KX702402.1	[32]
	Indonesia/2010	AB678695.1	[33]
	China/2012	KC488650.1	Direct submission
	Malaysia/2006	FN295484.2	[34]
	Thailand/1988	HM045789.1	[18]
	India/1963	HM045813.1	[18]
ECSA *	Angola/1962	HM045823.1	[18]
	Uganda/1982	HM045812.1	[18]
	Central African Republic/1978	HM045822.1	[18]
	Bahia/2014	KP164570.1	[17]
West African	Nigeria/1965	HM045807.1	[18]
	Senegal/2005	HM045817.1	[18]
	Ivory Coast/1993	HM045820.1	[18]

* ECSA: East-Central-South African genotype.

**Table 2:** Clinical, epidemiological, and demographic distribution of CHIKV and dengue virus (DENV) monoinfections and coinfections in Amapá State in 2014 and 2015.

	Monoinfections (%)			Coinfections (%)		*p*-Value
	CHIKV (*n* = 51)	DENV-1 (*n* = 7)	DENV-4 (*n* = 1)	CHIKV/DENV-1 (*n* = 1)	CHIKV/DENV-4 (*n* = 1)	
Municipality						
Macapá	5 (9.8)	3 (42.8)	1/1 (100)	1 (100)	0	0.445
Oiapoque	45 (88.2)	4 (57.1)	0	0	1 (100)	
Porto Grande	1 (2.0)	0	0	0	0	
Gender						
Female	37 (72.5)	1 (14.3)	0	1 (100)	1 (100)	0.319
Male	14 (27.4)	6 (85.7)	1 (100)	0	0	
Age group (years old)						
≤10	3 (5.9)	0	1 (100)	0	0	0.04
11–15	5 (9.8)	1 (14.3)	0	0	0	
16–20	5 (9.8)	0	0	0	0	
21–25	2 (3.9)	0	0	0	0	
26–30	6 (11.8)	1 (14.3)	0	0	0	
31–35	2 (3.9)	2 (28.6)	0	0	1 (100)	
36–40	4 (7.8)	2 (28.6)	0	0	0	
41–45	3 (5.9)	0	0	1 (100)	0	
46–50	7 (13.7)	0	0	0	0	
51–55	2 (3.9)	0	0	0	0	
56–60	0	1 (14.3)	0	0	0	
61–65	2 (3.9)	0	0	0	0	
>65	0	0	0	0	0	
Not informed	10 (19.6)	0	0	0	0	
Clinical manifestations						
Arthralgia	40 (78.4)	5 (71.4)	1 (100)		1 (100)	0.374
Headache	34 (66.7)	5 (71.4)	1 (100)		1 (100)	0.625
Diarrhea	5 (9.8)	0	0		0	0.585
Abdominal pain	1 (2.0)	1 (14.3)	0		0	0.685
Retro-Orbital pain	3 (5.9)	1 (14.3)	0		0	0.588
Edema	6 (11.8)	0	0		1 (100)	0.237
Exanthema	18 (35.3)	1 (14.3)	1 (100)		0	0.303
Fever	44 (86.3)	5 (71.4)	1 (100)		1 (100)	0.205
Myalgia	30 (58.8)	3 (42.8)	1 (100)		0	0.105
Petechiae	11 (21.6)	0	0		0	0.466
Cough	0	0	0		1 (100)	0.000
Vomiting	9 (17.6)	0	1 (100)		0	0.511

**Table 3:** Clinical, and demographic distribution of CHIKV and ZIKV monoinfections and CHIKV/ZIKV, CHIKV/DENV-4, and ZIKV/DENV-4 coinfections in Rio de Janeiro (RJ) during 2016.

	Monoinfections (%)		Coinfections (%)			*p* Value
	CHIKV (*n* = 40)	ZIKV (*n* = 9)	CHIKV/ZIKV (*n* = 16)	CHIKV/DENV-4 (*n* = 3)	ZIKV/DENV-4 (*n* = 1)	
Gender						
Female	22 (55.0)	4 (44.4)	10 (62.5)	3 (100)	1 (100)	0.196
Male	18 (45.0)	5 (55.5)	6 (37.5)	0	0	
Age group (years old)						
11–14	0	1 (11.1)	0	0	0	0.152
16–20	1 (2.5)	0	1 (6.2)	1 (33.3)	0	
21–25	1 (2.5)	0	1 (6.2)	1 (33.3)	0	
26–30	9 (22.5)	2 (22.2)	1 (6.2)	0	0	
31–35	3 (7.5)	0	1 (6.2)	0	0	
36–40	2 (5.0)	2 (22.2)	1 (6.2)	0	0	
41–45	4 (10.0)	2 (22.2)	4 (25.0)	0	0	
46–50	2 (5.0)	1(11.1)	1(6.2)	0	0	
51–55	6 (15.0)	0	1 (6.2)	0	1 (100)	
56–60	5 (12.5)	0	1 (6.2)	1 (33.3)	0	
61–65	2 (5.0)	1 (11.1)	2 (12.5)	0	0	
>65	5 (12.5)	0	2 (12.5)	0	0	
Clinical manifestations						
Anorexia	26 (65.0)	6 (66.6)	14 (87.5)	0	0	0.803
Arthralgia	36 (90.0)	7 (77.7)	15 (93.7)	3 (100)	1 (100)	0.565
Arthritis	18 (45.0)	2 (22.2)	6 (37.5)	0	1 (100)	0.042
Chills	29 (72.5)	4 (4.4)	10 (62.5)	2 (66.6)	1 (100)	0.828
Headache	31 (77.5)	9 (100)	13 (81.2)	2 (66.6)	1 (100)	0.668
Coryza	5 (12.5)	1 (11.1)	3 (18.7)	0	0	0.515
Abdominal pain	5 (12.5)	0	4 (25.0)	0	0	0.515
Sore throat	6 (15.0)	2 (22.2)	3 (18.7)	1 (33.3)	1 (100)	0.407
Retro-Orbital Pain	17 (42.5)	2 (22.2)	5 (31.2)	1 (33.3)	0	0.756
Edema	20 (50.0)	1 (11.1)	6 (37.5)	2 (66.6)	0	0.489
Epigastralgia	2 (5.0)	1 (11.1)	1 (6.2)	0	0	0.692
Exanthema	23 (57.5)	4 (4.4)	6 (37.5)	1 (33.3)	1 (100)	0.416
Fever	34 (85.0)	6 (66.6)	11 (68.7)	2 (66.6)	1 (100)	0.407
Conjunctival hyperemia	15 (37.5)	3 (33.3)	7 (43.7)	0	0	0.189
Low back pain	25 (62.5)	4 (4.4)	12 (75.0)	3 (100)	1 (100)	0.189
Myalgia	37 (92.5)	9 (100)	14 (87.5)	2 (66.6)	1 (100)	0.137
Nausea	19 (47.5)	5 (55.5)	4 (25.0)	2 (66.6)	1 (100)	0.522
Paresthesia	2 (5.0)	2 (22.2)	1 (6.2)	0	0	0.692
Hipovolemia	1 (2.5)	0	0	0	0	0.782
Prostration	33 (82.5)	8 (8.8)	13 (81.2)	2 (66.6)	1 (100)	0.497
Itching	18 (45.0)	4 (4.4)	6 (37.5)	2 (66.6)	1 (100)	0.608
Dizziness/Vertigo	9 (22.5)	2 (22.2)	8 (50.0)	1 (33.3)	0	0.668
Cough	2 (5.0)	2 (22.2)	2 (12.5)	0	0	0.692
Vomiting	5 (12.5)	1 (11.1)	4/ (25.0)	1 (33.3)	0	0.315

**Table 4:** The CHIKV strains from Amapá (*n* = 17) and Rio de Janeiro (*n* = 10) used in this study for partial E1 gene (375 nucleotides) sequencing, 2014 to 2016, Brazil.

ID Samples	Year	Country Region: City/State	Origin of Strain	Genbank Accession Number	Reference
18/BR/AP/2015	2015	North: Macapá/AP	Serum	MK105475	This study
46/BR/AP/2015	2015	North: Macapá/AP	Whole blood	MK105476	This study
47/BR/AP/2014	2014	North: Oiapoque/AP	Whole blood	MK105477	This study
85/BR/AP/2014	2014	North: Oiapoque/AP	Whole blood	MK105478	This study
91/BR/AP/2014	2014	North: Oiapoque/AP	Whole blood	MK105479	This study
111/BR/AP/2014	2014	North: Oiapoque/AP	Whole blood	MK105480	This study
112/BR/AP/2014	2014	North: Oiapoque/AP	Whole blood	MK105481	This study
396/BR/AP/2014	2014	North: Oiapoque/AP	Serum	MK105482	This study
429/BR/AP/2014	2014	North: Oiapoque/AP	Serum	MK105483	This study
430/BR/AP/2014	2014	North: Oiapoque/AP	Serum	MK105484	This study
442/BR/AP/2014	2014	North: Oiapoque/AP	Serum	MK105485	This study
443/BR/AP/2014	2014	North: Oiapoque/AP	Serum	MK105486	This study
444/BR/AP/2014	2014	North: Oiapoque/AP	Serum	MK105487	This study
446/BR/AP/2014	2014	North: Oiapoque/AP	Serum	MK105488	This study
447/BR/AP/2014	2014	North: Oiapoque/AP	Serum	MK105489	This study
452/BR/AP/2014	2014	North: Oiapoque/AP	Serum	MK105490	This study
453/BR/AP/2014	2014	North: Oiapoque/AP	Serum	MK105491	This study
ASR24/BR/RJ/2016	2016	Southeast: Rio de Janeiro/RJ	Plasma	KX966400.1	[35]
EFSG07/BR/RJ/2016	2016	Southeast: Rio de Janeiro/RJ	Plasma	KX966401.1	[35]
EMC18/BR/RJ/2016	2016	Southeast: Rio de Janeiro/RJ	Plasma	KX966402.1	[35]
GP18/BR/RJ/2016	2016	Southeast: Rio de Janeiro/RJ	Plasma	KX966403.1	[35]
LATC25/BR/RJ/2016	2016	Southeast: Rio de Janeiro/RJ	Plasma	KX966404.1	[35]
LCSS29/BR/RJ/2016	2016	Southeast: Rio de Janeiro/RJ	Plasma	KX966405.1	[35]
MMT16/BR/RJ/2016	2016	Southeast: Rio de Janeiro/RJ	Plasma	KX966406.1	[35]
SHCV30/BR/RJ/2016	2016	Southeast: Rio de Janeiro/RJ	Plasma	KX966407.1	[35]
SMSG20/BR/RJ/2016	2016	Southeast: Rio de Janeiro/RJ	Plasma	KX966408.1	[35]
JOM22/BR/RJ/2016	2016	Southeast: Rio de Janeiro/RJ	Plasma	KX966409.1	[35]

ID: Identification; AP: Amapá; BR: Brazil; RJ: Rio de Janeiro.

## References

[1] Lum F.M., Ng L.F. (2015). Cellular and molecular mechanisms of chikungunya pathogenesis. Antiviral Res..

[2] Solignat M., Gay B., Higgs S., Briant L., Devaux C. (2009). Replication cycle of chikungunya: A re-emerging arbovirus. Virology.

[3] Strauss J.H., Strauss E.G. (1994). The alphaviruses: Gene expression, replication, and evolution. Microbiol. Rev..

[4] Lo Presti A., Cella E., Angeletti S., Ciccozzi M. (2016). Molecular epidemiology, evolution and phylogeny of Chikungunya virus: An updating review. Infect. Genet. Evol. J. Mol. Epidemiol. Evol. Genet. Infect. Dis..

[5] (2014). MS, (Ministério da Saúde) Preparação e Resposta à Introdução do Vírus Chikungunya No Brasil. http://bvsms.saude.gov.br/bvs/publicacoes/preparacao_resposta_virus_chikungunya_brasil.pdf.

[6] Rougeron V., Sam I.-C., Caron M., Nkoghe D., Leroy E., Roques P. (2015). Chikungunya, a paradigm of neglected tropical disease that emerged to be a new health global risk. J. Clin. Virol..

[7] Diallo M., Thonnon J., Traore-Lamizana M., Fontenille D. (1999). Vectors of Chikungunya virus in Senegal: Current data and transmission cycles. Am. J. Trop. Med. Hyg..

[8] Gilotra S.K., Shah K.V. (1967). Laboratory studies on transmission of Chikungunya virus by mosquitoes. Am. J. Epidemiol..

[9] Carey D.E. (1971). Chikungunya and dengue: A case of mistaken identity?. J. Hist. Med. Allied Sci..

[10] Simon F., Parola P., Grandadam M., Fourcade S., Oliver M., Brouqui P., Hance P., Kraemer P., Ali Mohamed A., de Lamballerie X. (2007). Chikungunya infection: An emerging rheumatism among travelers returned from Indian Ocean islands. Report of 47 cases. Medicine.

[11] Lee V.J., Chow A., Zheng X., Carrasco L.R., Cook A.R., Lye D.C., Ng L.-C., Leo Y.-S. (2012). Simple clinical and laboratory predictors of Chikungunya versus dengue infections in adults. PLoS Negl. Trop. Dis..

[12] Fourie E.D., Morrison J.G. (1979). Rheumatoid arthritic syndrome after chikungunya fever. S. Afr. Med. J. Suid-Afr. Tydskr. Vir Geneeskd..

[13] Thiberville S.-D., Boisson V., Gaudart J., Simon F., Flahault A., de Lamballerie X. (2013). Chikungunya fever: A clinical and virological investigation of outpatients on Reunion Island, South-West Indian Ocean. PLoS Negl. Trop. Dis..

[14] Sissoko D., Malvy D., Ezzedine K., Renault P., Moscetti F., Ledrans M., Pierre V. (2009). Post-epidemic Chikungunya disease on Reunion Island: Course of rheumatic manifestations and associated factors over a 15-month period. PLoS Negl. Trop. Dis..

[15] Burt F.J., Rolph M.S., Rulli N.E., Mahalingam S., Heise M.T. (2012). Chikungunya: A re-emerging virus. Lancet Lond. Engl..

[16] Simizu B., Yamamoto K., Hashimoto K., Ogata T. (1984). Structural proteins of Chikungunya virus. J. Virol..

[17] Nunes M.R., Faria N.R., de Vasconcelos J.M., Golding N., Kraemer M.U., de Oliveira L.F., Azevedo R.S., da Silva D.E., da Silva E.V., da Silva S.P. (2015). Emergence and potential for spread of Chikungunya virus in Brazil. BMC Med..

[18] Volk S.M., Chen R., Tsetsarkin K.A., Adams A.P., Garcia T.I., Sall A.A., Nasar F., Schuh A.J., Holmes E.C., Higgs S. (2010). Genome-scale phylogenetic analyses of chikungunya virus reveal independent emergences of recent epidemics and various evolutionary rates. J. Virol..

[19] Tsetsarkin K.A., Chen R., Sherman M.B., Weaver S.C. (2011). Chikungunya virus: Evolution and genetic determinants of emergence. Curr. Opin. Virol..

[20] Honório N.A., Câmara D.C., Calvet G.A., Brasil P. (2015). Chikungunya: An arbovirus infection in the process of establishment and expansion in Brazil. Cad. Saude Publ..

[21] Brasil P., Calvet G.A., Siqueira A.M., Wakimoto M., de Sequeira P.C., Nobre A., Quintana M.S., Mendonça M.C., Lupi O., de Souza R.V. (2016). Zika Virus Outbreak in Rio de Janeiro, Brazil: Clinical Characterization, Epidemiological and Virological Aspects. PLoS Negl. Trop. Dis..

[22] SVS Secretaria de Vigilância em Saúde—Ministério da Saúde Monitoramento dos Casos de Dengue, Febre de Chikungunya e Febre pelo vírus Zika até a Semana Epidemiológica 52, 2017. http://portalarquivos2.saude.gov.br/images/pdf/2018/janeiro/23/Boletim-2018-001-Dengue.pdf.

[23] Lanciotti R.S., Kosoy O.L., Laven J.J., Panella A.J., Velez J.O., Lambert A.J., Campbell G.L. (2007). Chikungunya virus in US travelers returning from India, 2006. Emerg. Infect. Dis..

[24] Mohan A., Kiran D.H., Manohar I.C., Kumar D.P. (2010). Epidemiology, clinical manifestations, and diagnosis of Chikungunya fever: Lessons learned from the re-emerging epidemic. Indian J. Dermatol..

[25] Pialoux G., Gaüzère B.-A., Jauréguiberry S., Strobel M. (2007). Chikungunya, an epidemic arbovirosis. Lancet Infect. Dis..

[26] Lanciotti R.S., Calisher C.H., Gubler D.J., Chang G.J., Vorndam A.V. (1992). Rapid detection and typing of dengue viruses from clinical samples by using reverse transcriptase-polymerase chain reaction. J. Clin. Microbiol..

[27] Johnson B.W., Russell B.J., Lanciotti R.S. (2005). Serotype-specific detection of dengue viruses in a fourplex real-time reverse transcriptase PCR assay. J. Clin. Microbiol..

[28] Lanciotti R.S., Kosoy O.L., Laven J.J., Velez J.O., Lambert A.J., Johnson A.J., Stanfield S.M., Duffy M.R. (2008). Genetic and serologic properties of Zika virus associated with an epidemic, Yap State, Micronesia, 2007. Emerg. Infect. Dis..

[29] De Morais Bronzoni R.V., Baleotti F.G., Ribeiro Nogueira R.M., Nunes M., Moraes Figueiredo L.T. (2005). Duplex reverse transcription-PCR followed by nested PCR assays for detection and identification of Brazilian alphaviruses and flaviviruses. J. Clin. Microbiol..

[30] Collao X., Negredo A.I., Cano J., Tenorio A., Ory F., Benito A., Masia M., Sánchez-Seco M.P. (2010). Different lineages of Chikungunya virus in Equatorial Guinea in 2002 and 2006. Am. J. Trop. Med. Hyg..

[31] Conteville L.C., Zanella L., Marín M.A., Filippis A.M., Nogueira R.M., Vicente A.C., Mendonça M.C. (2016). Phylogenetic analyses of chikungunya virus among travelers in Rio de Janeiro, Brazil, 2014–2015. Mem. Inst. Oswaldo Cruz.

[32] White S.K., Morris J.G., Elbadry M.A., Beau De Rochars V.M., Okech B.A., Lednicky J.A. (2017). Complete Genome Sequences of Chikungunya Viruses Isolated from Plasma Specimens Collected from Haitians in 2014. Genome Announc..

[33] Mulyatno K.C., Susilowati H., Yamanaka A., Soegijanto S., Konishi E. (2012). Primary isolation and phylogenetic studies of Chikungunya virus from Surabaya, Indonesia. Jpn. J. Infect. Dis..

[34] Sam I.-C., Loong S.-K., Michael J.C., Chua C.-L., Wan Sulaiman W.Y., Vythilingam I., Chan S.-Y., Chiam C.-W., Yeong Y.-S., AbuBakar S. (2012). Genotypic and Phenotypic Characterization of Chikungunya Virus of Different Genotypes from Malaysia. PLoS ONE.

[35] Souza T.M., Azeredo E.L., Badolato-Corrêa J., Damasco P.V., Santos C., Petitinga-Paiva F., Nunes P.C., Barbosa L.S., Cipitelli M.C., Chouin-Carneiro T. (2017). First Report of the East-Central South African Genotype of Chikungunya Virus in Rio de Janeiro, Brazil. PLoS Curr..

[36] SVS/MS Boletim Epidemiológico (2015). Monitoramento dos casos de Dengue, Febre de Chikungunya e Doença Aguda pelo Vírus Zika até a Semana Epidemiológica 52 de 2015. Secr. Vigilância Em Saúde Minist. Saúde.

[37] Lima M.R., Nogueira R.M., Schatzmayr H.G., dos Santos F.B. (2010). Comparison of three commercially available dengue NS1 antigen capture assays for acute diagnosis of dengue in Brazil. PLoS Negl. Trop. Dis..

[38] Phommanivong V., Kanda S., Shimono T., Lamaningao P., Darcy A.W., Mishima N., Phaytanavanh B., Nishiyama T. (2016). Co-circulation of the dengue with chikungunya virus during the 2013 outbreak in the southern part of Lao PDR. Trop. Med. Health.

[39] Correia J.C., Barbosa R.M., Oliveira C.M., Albuquerque C.M. (2012). Residential characteristics aggravating infestation by Culex quinquefasciatus in a region of Northeastern Brazil. Rev. Saude Publ..

[40] Hoarau J.J., Jaffar Bandjee M.C., Krejbich Trotot P., Das T., Li-Pat-Yuen G., Dassa B., Denizot M., Guichard E., Ribera A., Henni T. (2010). Persistent chronic inflammation and infection by Chikungunya arthritogenic alphavirus in spite of a robust host immune response. J. Immunol..

[41] Riswari S.F., Ma’roef C.N., Djauhari H., Kosasih H., Perkasa A., Yudhaputri F.A., Artika I.M., Williams M., van der Ven A., Myint K.S. (2016). Study of viremic profile in febrile specimens of chikungunya in Bandung, Indonesia. J. Clin. Virol..

[42] Appassakij H., Khuntikij P., Kemapunmanus M., Wutthanarungsan R., Silpapojakul K. (2013). Viremic profiles in asymptomatic and symptomatic chikungunya fever: A blood transfusion threat?. Transfusion (Paris).

[43] Poo Y.S., Rudd P.A., Gardner J., Wilson J.A., Larcher T., Colle M.A., Le T.T., Nakaya H.I., Warrilow D., Allcock R. (2014). Multiple immune factors are involved in controlling acute and chronic chikungunya virus infection. PLoS Negl. Trop. Dis..

[44] Chow A., Her Z., Ong E.K., Chen J.M., Dimatatac F., Kwek D.J., Barkham T., Yang H., Rénia L., Leo Y.S. (2011). Persistent arthralgia induced by Chikungunya virus infection is associated with interleukin-6 and granulocyte macrophage colony-stimulating factor. J. Infect. Dis..

[45] Simon F., Javelle E., Oliver M., Leparc-Goffart I., Marimoutou C. (2011). Chikungunya virus infection. Curr. Infect. Dis. Rep..

[46] Gautret P., Simon F. (2016). Dengue, chikungunya and Zika and mass gatherings: What happened in Brazil, 2014. Travel Med. Infect. Dis..

[47] Azeredo E.L., Dos Santos F.B., Barbosa L.S., Souza T.M.A., Badolato-Corrêa J., Sánchez-Arcila J.C., Nunes P.C.G., de-Oliveira-Pinto L.M., de Filippis A.M., Dal Fabbro M. (2018). Clinical and Laboratory Profile of Zika and Dengue Infected Patients: Lessons Learned from the Co-circulation of Dengue, Zika and Chikungunya in Brazil. PLoS Curr..

[48] Sardi S.I., Somasekar S., Naccache S.N., Bandeira A.C., Tauro L.B., Campos G.S., Chiu C.Y. (2016). Coinfections of Zika and Chikungunya Viruses in Bahia, Brazil, Identified by Metagenomic Next-Generation Sequencing. J. Clin. Microbiol..

[49] Villamil-Gómez W.E., Rodríguez-Morales A.J., Uribe-García A.M., González-Arismendy E., Castellanos J.E., Calvo E.P., Álvarez-Mon M., Musso D. (2016). Zika, dengue, and chikungunya co-infection in a pregnant woman from Colombia. Int. J. Infect. Dis..

[50] Essackjee K., Goorah S., Ramchurn S.K., Cheeneebash J., Walker-Bone K. (2013). Prevalence of and risk factors for chronic arthralgia and rheumatoid-like polyarthritis more than 2 years after infection with chikungunya virus. Postgrad. Med. J..

[51] SVS/MS Monitoramento dos casos de Dengue e febre de Chikungunya até a Semana Epidemiológica (SE) 53 de 2014. http://portalarquivos2.saude.gov.br/images/pdf/2015/janeiro/19/2015-002---BE-at---SE-53.pdf.

[52] Borgherini G., Poubeau P., Jossaume A., Gouix A., Cotte L., Michault A., Arvin-Berod C., Paganin F. (2008). Persistent arthralgia associated with chikungunya virus: A study of 88 adult patients on reunion island. Clin. Infect. Dis..

[53] (2015). MS, (Ministério da Saúde) Febre de Chikungunya: Manejo clínico. http://bvsms.saude.gov.br/bvs/publicacoes/febre_chikungunya_manejo_clinico.pdf.

[54] Bandyopadhyay D., Ghosh S.K. (2008). Mucocutaneous features of Chikungunya fever: A study from an outbreak in West Bengal, India. Int. J. Dermatol..

[55] Ali Ou Alla S., Combe B. (2011). Arthritis after infection with Chikungunya virus. Best Pract. Res. Clin. Rheumatol..

[56] Kucharz E.J., Cebula-Byrska I. (2012). Chikungunya fever. Eur. J. Intern. Med..

[57] Malvy D., Ezzedine K., Mamani-Matsuda M., Autran B., Tolou H., Receveur M.-C., Pistone T., Rambert J., Moynet D., Mossalayi D. (2009). Destructive arthritis in a patient with chikungunya virus infection with persistent specific IgM antibodies. BMC Infect. Dis..

[58] De Andrade D.C., Jean S., Clavelou P., Dallel R., Bouhassira D. (2010). Chronic pain associated with the Chikungunya Fever: Long lasting burden of an acute illness. BMC Infect. Dis..

[59] Larrieu S., Pouderoux N., Pistone T., Filleul L., Receveur M.-C., Sissoko D., Ezzedine K., Malvy D. (2010). Factors associated with persistence of arthralgia among Chikungunya virus-infected travellers: Report of 42 French cases. J. Clin. Virol..

[60] Chopra A., Anuradha V., Ghorpade R., Saluja M. (2012). Acute Chikungunya and persistent musculoskeletal pain following the 2006 Indian epidemic: A 2-year prospective rural community study. Epidemiol. Infect..

[61] Schilte C., Staikowsky F., Staikovsky F., Couderc T., Madec Y., Carpentier F., Kassab S., Albert M.L., Lecuit M., Michault A. (2013). Chikungunya virus-associated long-term arthralgia: A 36-month prospective longitudinal study. PLoS Negl. Trop. Dis..

[62] Couturier E., Guillemin F., Mura M., Léon L., Virion J.-M., Letort M.-J., De Valk H., Simon F., Vaillant V. (2012). Impaired quality of life after chikungunya virus infection: A 2-year follow-up study. Rheumatol. Oxf. Engl..

[63] Yaseen H.M., Simon F., Deparis X., Marimoutou C. (2014). Identification of initial severity determinants to predict arthritis after chikungunya infection in a cohort of French gendarmes. BMC Musculoskelet. Disord..

[64] Parreira R., Centeno-Lima S., Lopes A., Portugal-Calisto D., Constantino A., Nina J. (2014). Dengue virus serotype 4 and chikungunya virus coinfection in a traveller returning from Luanda, Angola, January 2014. Eurosurveillance.

[65] Teixeira M.G., Andrade A.M., Costa M.C.N., Castro J.N., Oliveira F.L., Goes C.S., Maia M., Santana E.B., Nunes B.T., Vasconcelos P.F. (2015). East/Central/South African Genotype Chikungunya Virus, Brazil, 2014. Emerg. Infect. Dis..

[66] Tsetsarkin K.A., Vanlandingham D.L., McGee C.E., Higgs S. (2007). A single mutation in chikungunya virus affects vector specificity and epidemic potential. PLoS Pathog..

[67] Tsetsarkin K.A., Weaver S.C. (2011). Sequential adaptive mutations enhance efficient vector switching by Chikungunya virus and its epidemic emergence. PLoS Pathog..

[68] Kariuki Njenga M., Nderitu L., Ledermann J.P., Ndirangu A., Logue C.H., Kelly C.H.L., Sang R., Sergon K., Breiman R., Powers A.M. (2008). Tracking epidemic Chikungunya virus into the Indian Ocean from East Africa. J. Gen. Virol..

[69] Niyas K.P., Abraham R., Unnikrishnan R.N., Mathew T., Nair S., Manakkadan A., Issac A., Sreekumar E. (2010). Molecular characterization of Chikungunya virus isolates from clinical samples and adult Aedes albopictus mosquitoes emerged from larvae from Kerala, South India. Virol. J..

[70] Sumathy K., Ella K.M. (2012). Genetic diversity of Chikungunya virus, India 2006–2010: Evolutionary dynamics and serotype analyses. J. Med. Virol..

[71] Agarwal A., Sharma A.K., Sukumaran D., Parida M., Dash P.K. (2016). Two novel epistatic mutations (E1:K211E and E2:V264A) in structural proteins of Chikungunya virus enhance fitness in Aedes aegypti. Virology.

